# How Marital Status Shapes Grandparenting Children With Disabilities

**DOI:** 10.1093/geroni/igab046.929

**Published:** 2021-12-17

**Authors:** Madonna Harrington Meyer

**Affiliations:** Sociology, Syracuse, New York, United States

## Abstract

How does marital status shape grandparent care work when grandchildren have disabilities? Based on 50 in-depth interviews with grandparents who provide various types of care for grandchildren with disabilities, we find that marital status shapes care work in three distinct ways: (1) Many who are married describe both grandparents working as a team to provide vital care; (2) Some who are married describe spouses, primarily grandfathers, who are either unable or unwilling to provide care; and (3) Many who are not married, primarily grandmothers, describe providing relatively high levels of care and support despite relatively low resources. While nearly all report a great deal of joy and satisfaction with their care work, those who are single, have greater care responsibilities, and fewer resources are more likely to report adverse social, emotional, physical, and financial impacts. More robust social policies could alleviate the impact of marital status on grandparent care work.

